# Supplementary material for: A modified rat model of 8 minutes asphyxial cardiac arrest and cardiopulmonary resuscitation
Source: PLoS One. 2025 Apr 29;20(4):e0322473. doi: 10.1371/journal.pone.0322473 (PMC12040107; doi:10.1371/journal.pone.0322473)

Raw image-HE images
Sham group cortex
S1

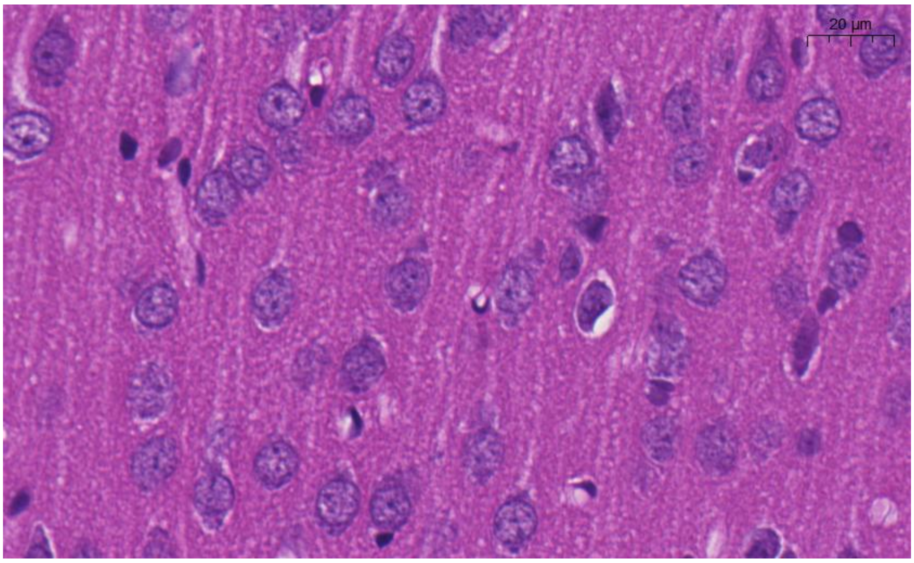

S2

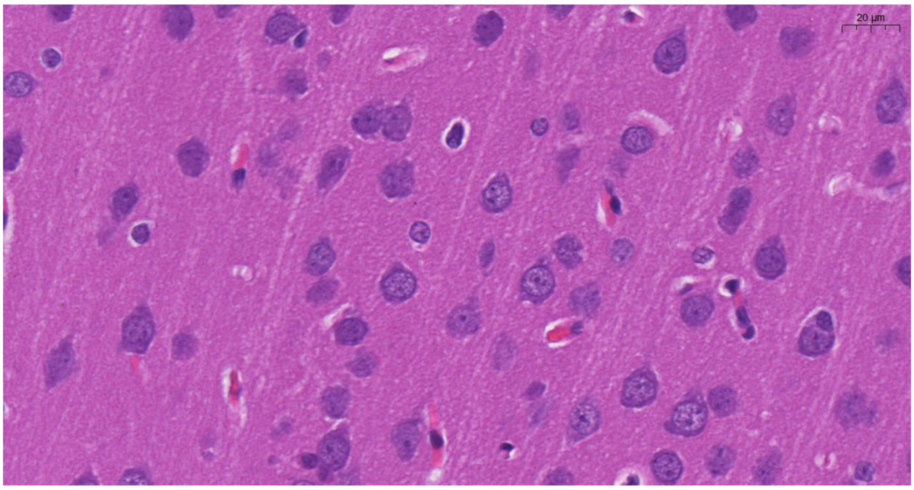

S3

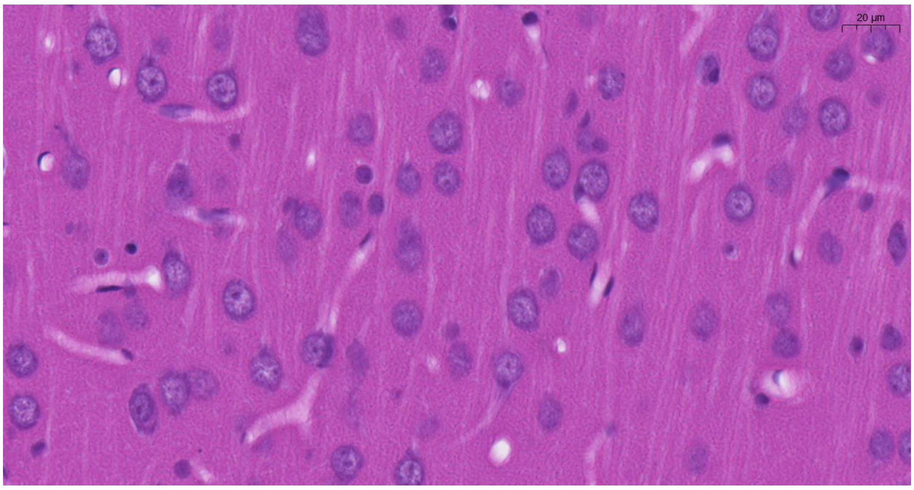

Sham group CA1

S1

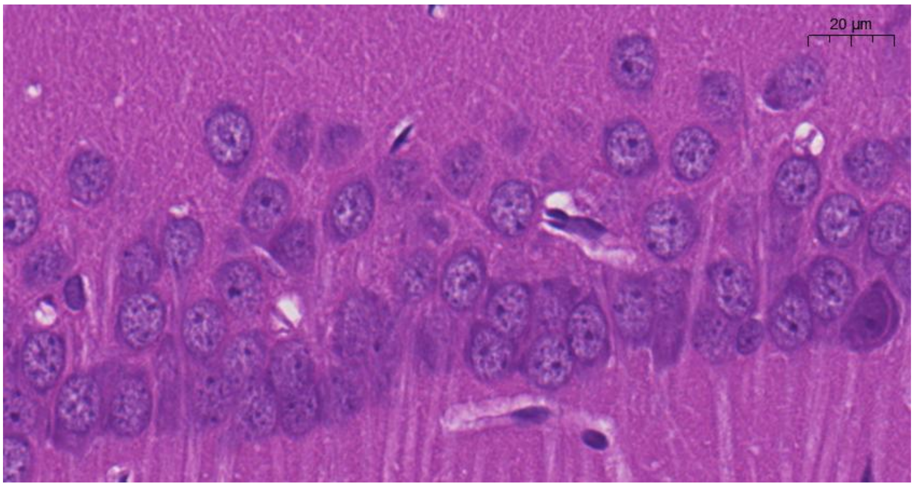

S2

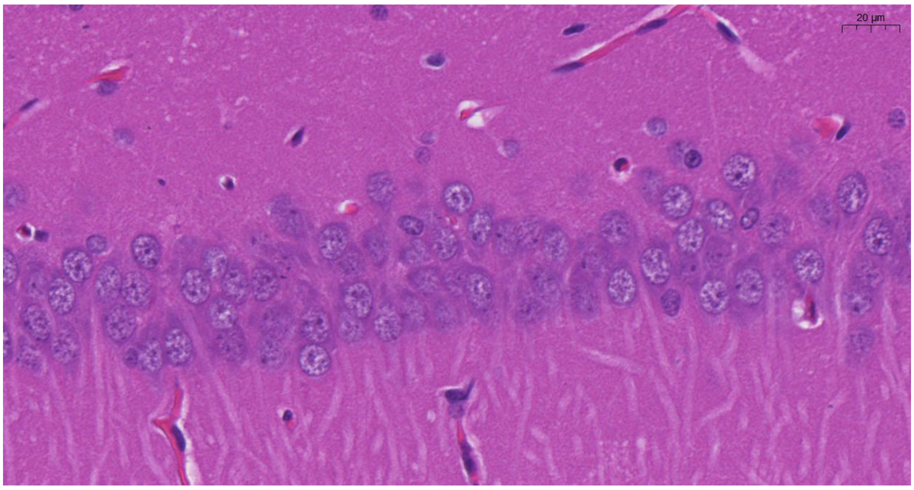

S3

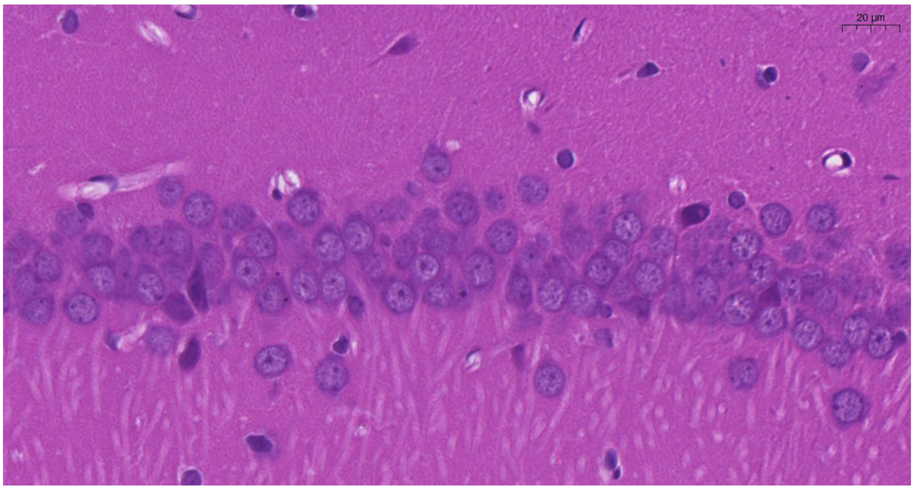

Sham group DG

S1

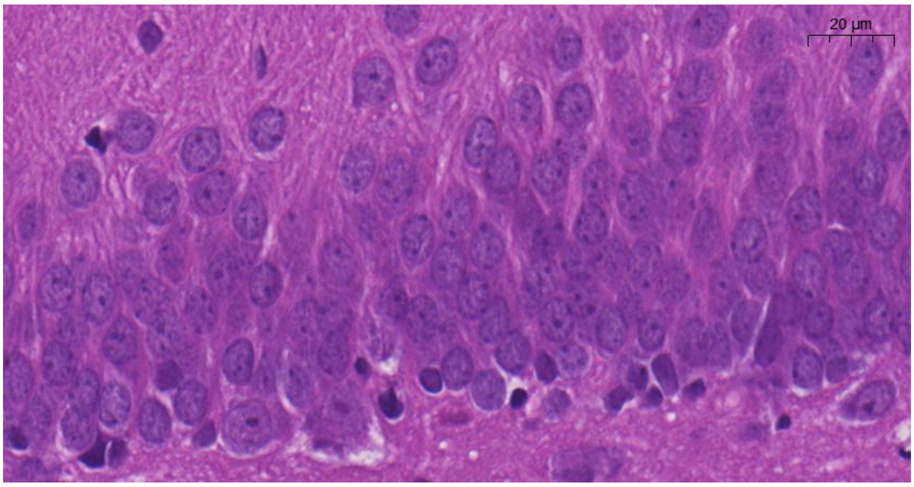

S2

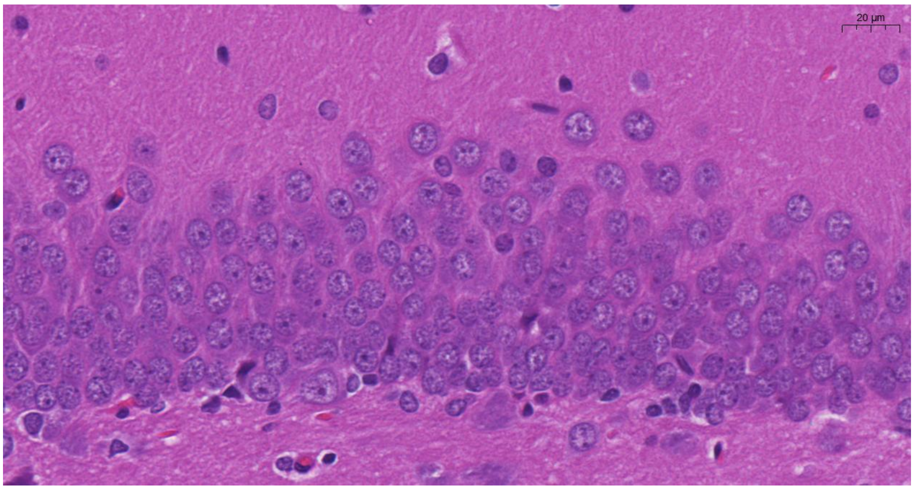

S3

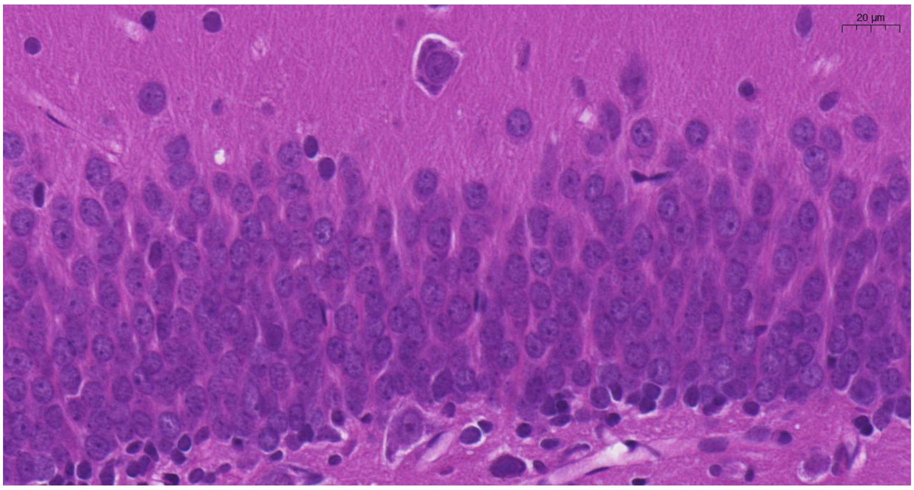

CA group Cortex

C1

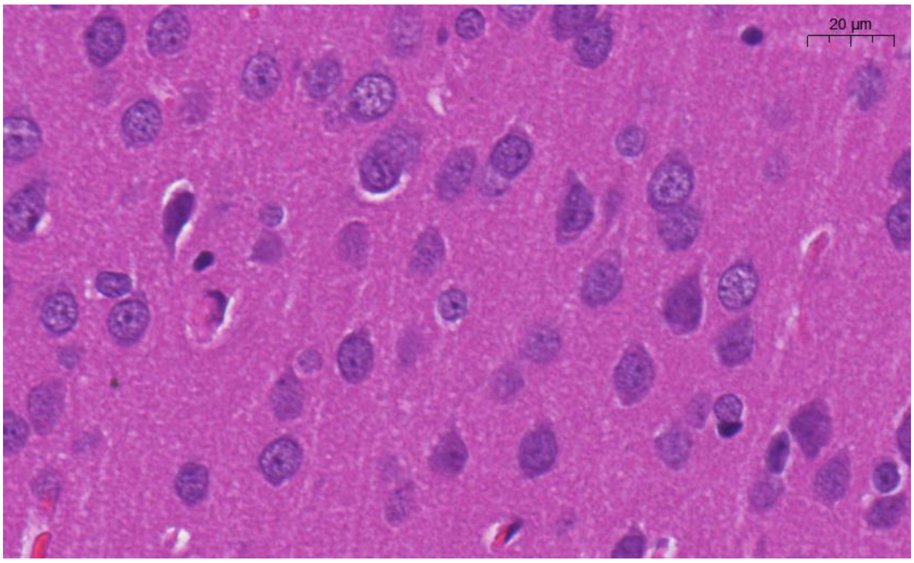

C2

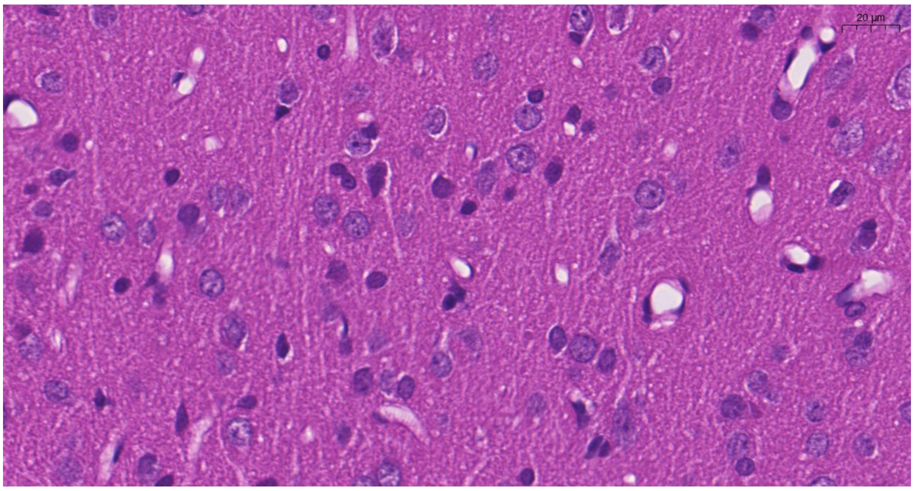

C3

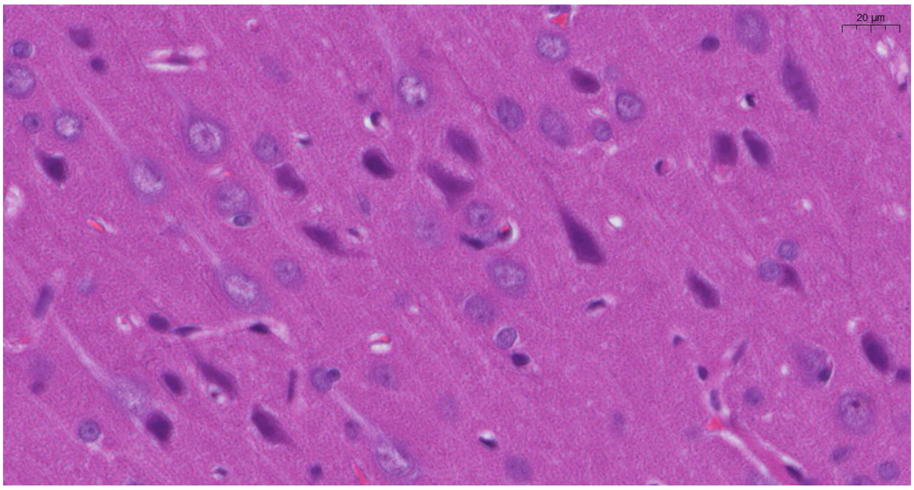

CA group CA1

C1

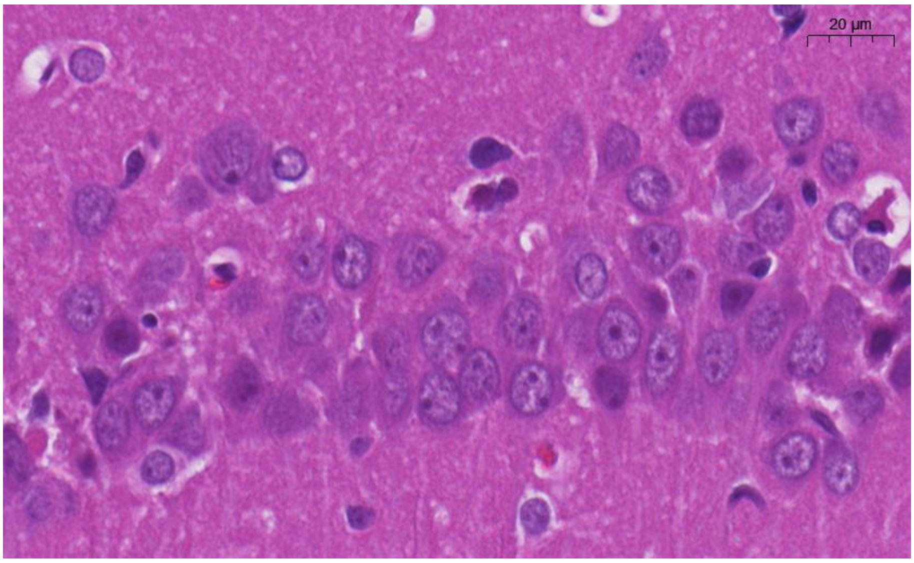

C2

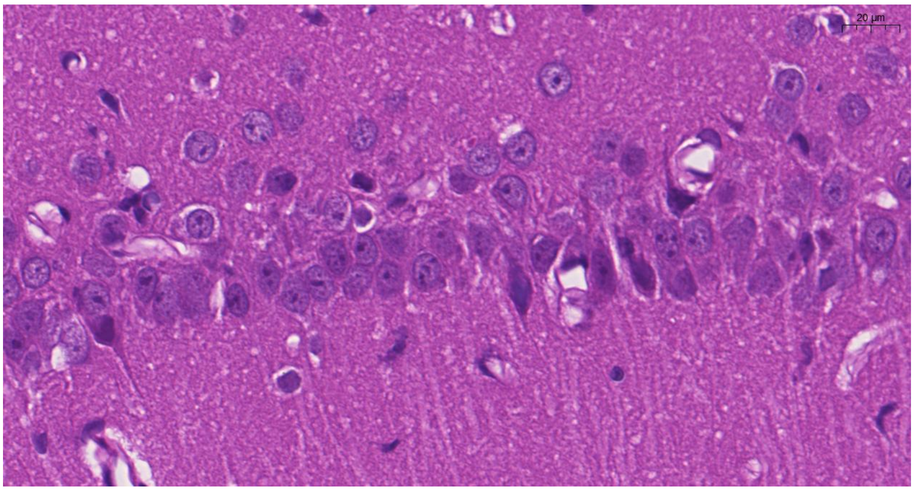

C3

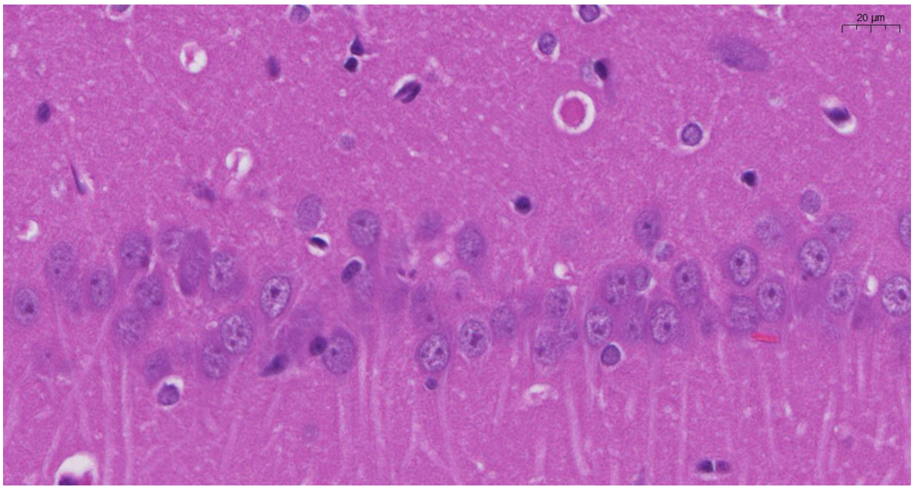

CA group DG

C1

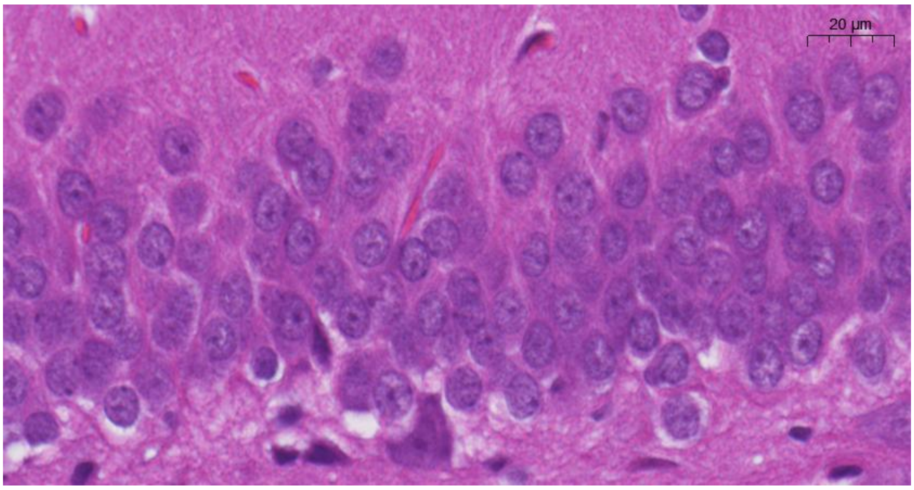

C2

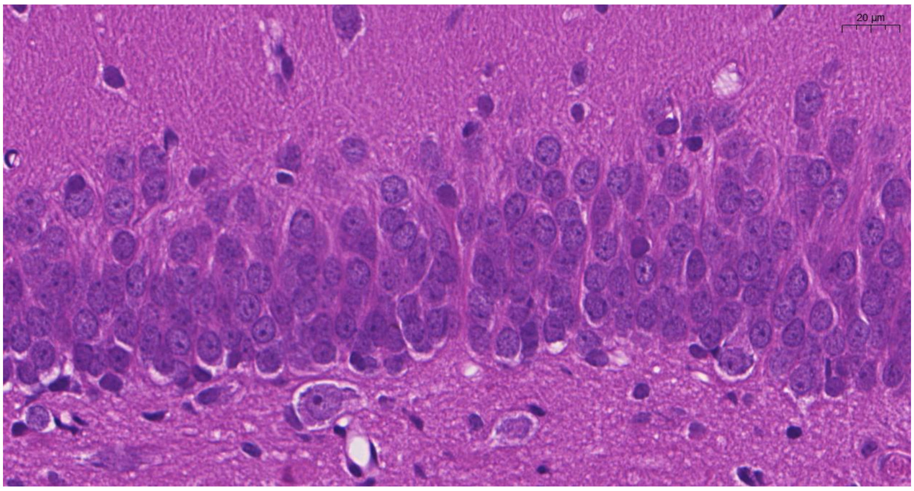

C3

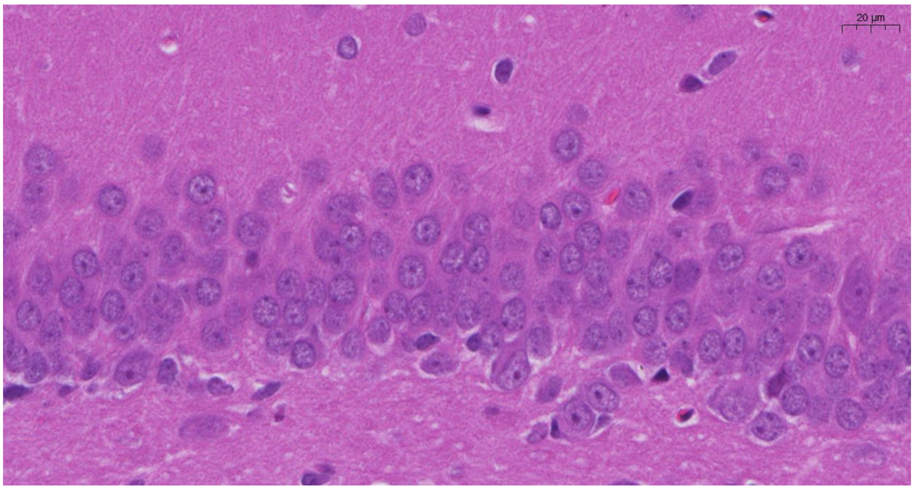

Supplement: S1 Image — HE images. (PDF) [file pone.0322473.s002.pdf]
