# Supplementary figures and images for: A modified rat model of 8 minutes asphyxial cardiac arrest and cardiopulmonary resuscitation
Source: PLoS One. 2025 Apr 29;20(4):e0322473. doi: 10.1371/journal.pone.0322473 (PMC12040107; doi:10.1371/journal.pone.0322473)

**Raw Image-TEM Image**

**Sham group**

**S1**

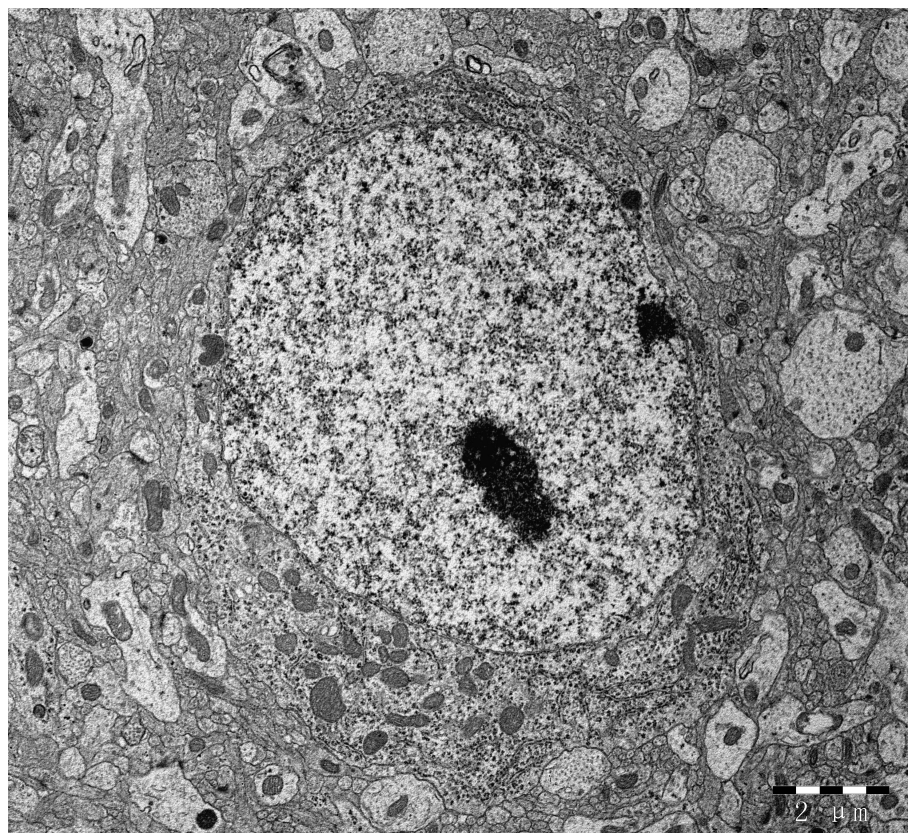

**S2**

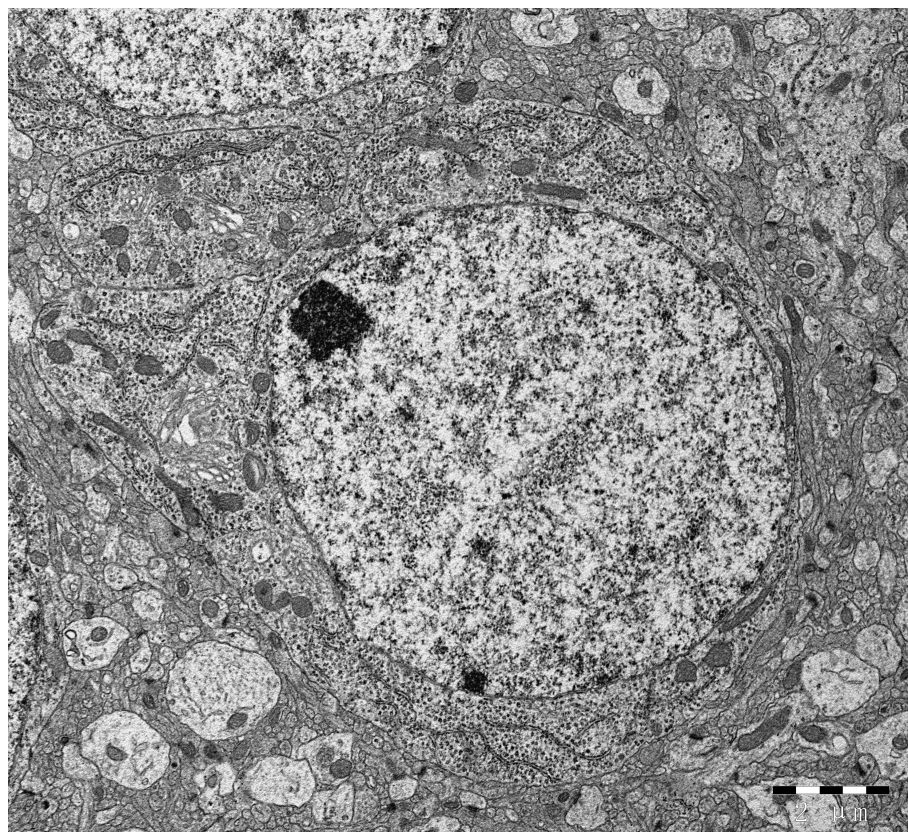

**S3**

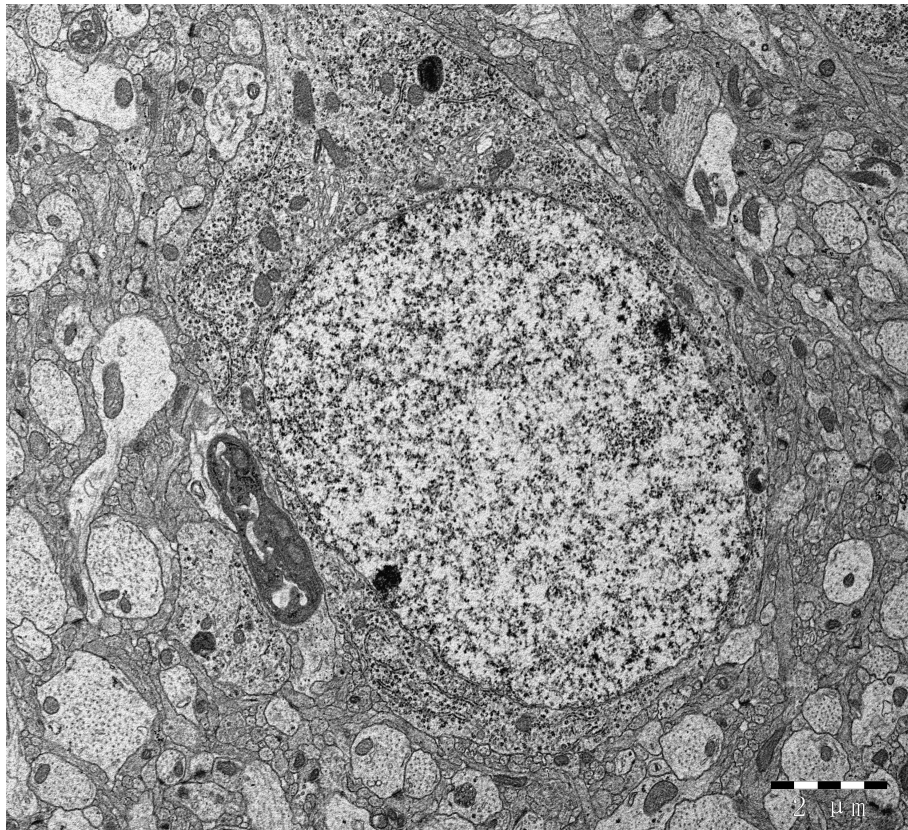

**CA group**

**C1**

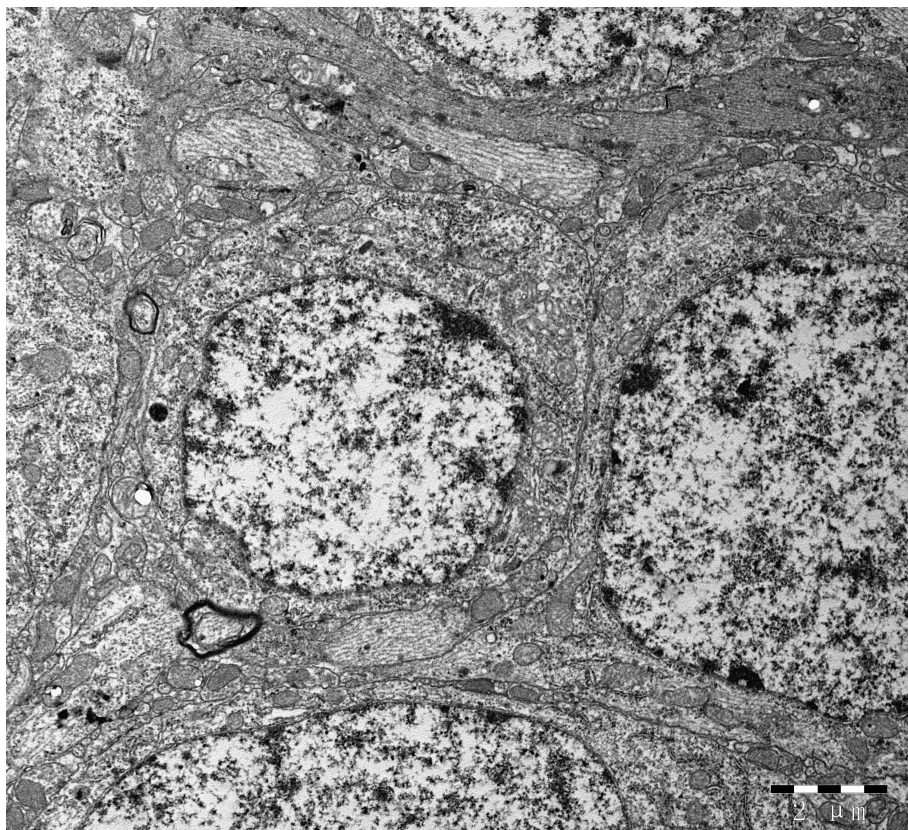

C2

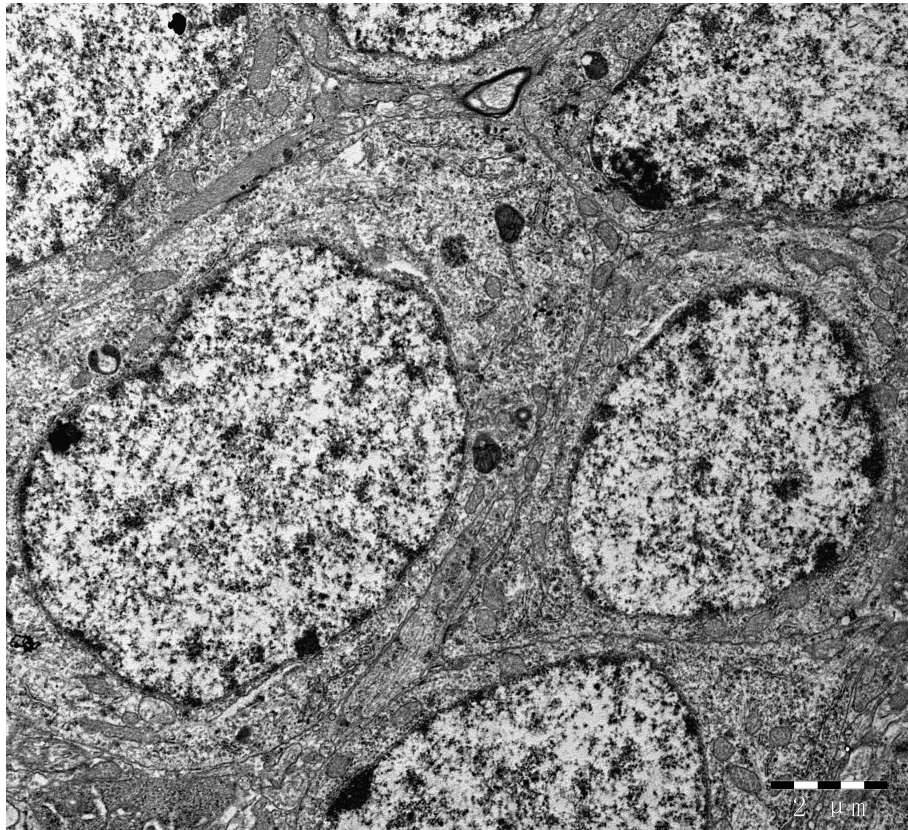

C3

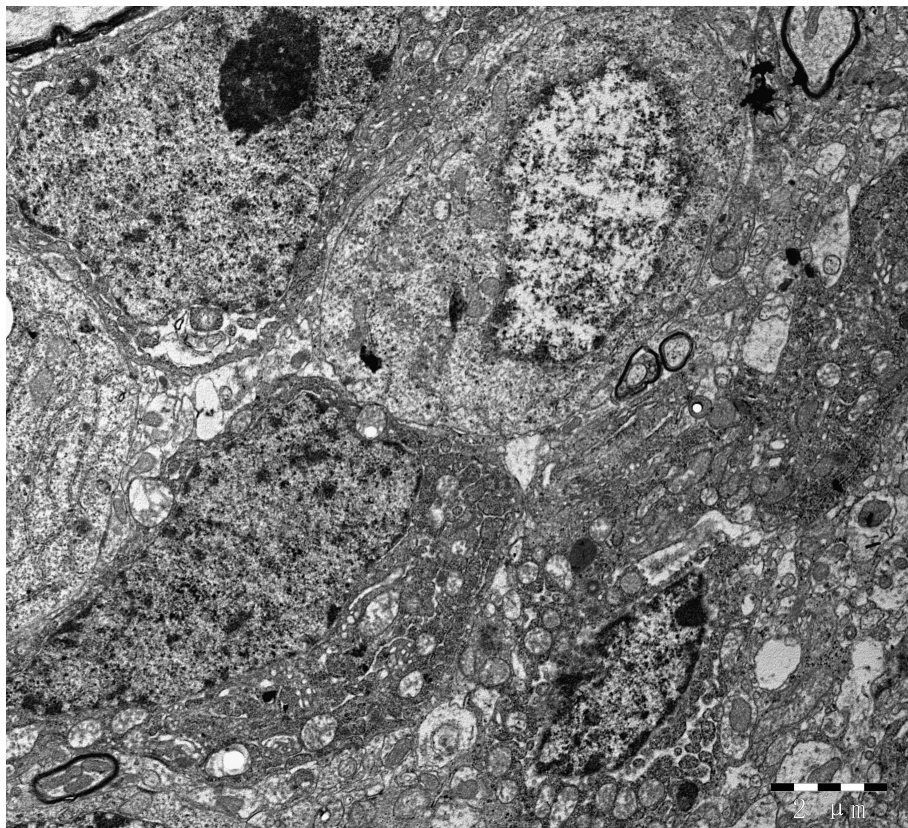

Supplement: S2 Image — TEM images. (PDF) [file pone.0322473.s003.pdf]
