# Supplementary material for: A modified rat model of 8 minutes asphyxial cardiac arrest and cardiopulmonary resuscitation
Source: PLoS One. 2025 Apr 29;20(4):e0322473. doi: 10.1371/journal.pone.0322473 (PMC12040107; doi:10.1371/journal.pone.0322473)
Supplement: Attachment — Submitted filename: Response to Reviewers.docx [file pone.0322473.s005.docx]

Dear Editors and Reviewers:

Thank you for the opportunity to revise our manuscript and address the reviewers’ comments. We appreciate the thorough evaluation by the academic editor and reviewers, and we have carefully revised the manuscript to incorporate their suggestions. Below, we provide a point-by-point response to all concerns raised.

Response to Academic Editor’s Comments

Editor’s Comment: [Address minor concerns raised by the reviewer].

Response: We thank the editor for this observation. We have discussed the issues raised by the reviewers in more detail in the revised manuscript and clearly marked the revisions. We supplemented the re-uploaded attachment with more detailed original neurobehavioral data and uploaded additional HE images and TEM images of animals in the PACE system.

Response to Reviewer 1’s Comments

Reviewer 1:

Opinion 1: [Please compare the results of this study with those reported by HHL Hendrickx (Resuscitation, 1984) and L Katz (JFRBM, 1995) in the discussion, and answer why the 72-hour survival rate was higher in the Katz’s study when a similar procedure was used.]

Response: We appreciate this suggestion. In the newly submitted manuscript, we discuss and contrast the original methods presented in the article by Hendrickx and Katz, which are marked in red under "Revised Manuscript with Track Changes." Regarding the difference in the 72-hour survival rate between our experiment and that of Katz, a certain elaboration has also been made in the discussion section of the revised manuscript. I summarize the possible reasons as follows:

①Use of sodium bicarbonate during cardiopulmonary resuscitation. To minimize the effect of drugs on blood gas results after ROSC, sodium bicarbonate was not used in our experiment, which may be one of the reasons for the lower ROSC rate and 72-hour survival rate compared to the Katz study.

②Post-ROSC longer pure oxygen mechanical ventilation. After ROSC, when the spontaneous breathing of the rats recovered to the extubation conditions, we implemented the weaning and extubation protocol, and did not use muscle relaxants to keep the rats on pure oxygen mechanical ventilation continuously. However, Katz's protocol maintained at least 1 hour of pure oxygen ventilation. The rapid extubation protocol without longer ICU monitoring may lead to an increase in the number of rats that died due to respiratory system dysfunction in the early stage of resuscitation.

③The difference in body weight of SD rats leads to different tolerance to hypoxia. We selected rats weighing 250-300g, which were in the early adult stage. Katz selected rats weighing 350-400g. Adult rats with lower body weight and younger age have more active basal metabolism, higher oxygen consumption, faster lactate accumulation during hypoxia, are prone to acidosis, and have poorer tolerance to hypoxia in brain tissues [M J Durkot（Aviat Space Environ Med,1986）,M A Holliday(Pediatr Res,1967)].

To sum up, in our experimental results, the ROSC rate and 72-hour survival rate of rats were lower than those observed in Katz's study. However, based on the comprehensive neurological function score, behavior, and pathological results, 72 hours after resuscitation, the neurological function damage in our rats was more severe compared to that in Katz's rats. The neurological function damage in Katz's experiment was mild at 72 hours. We consider that more severe neurological function damage in the early stage of resuscitation may be more representative of the clinical situation.

Opinion 2: [The supplemental file is missing primary data for neurological outcomes presented in Figure 7C and 7D.]

Response: We agree with the reviewer’s concern. We uploaded all the original data of SD rats in the research in the previous attachment, including the data of "mNSS score and Chimney test in Neurobehavioral experiments". This time, we supplemented the scores of each part within the total score of mNSS score in the original data of SD rats. Their locations can be found in the newly submitted Excel file "SDrat - raw data" .

Opinion 3: [It is recommended to supplement additional H&E staining images of more animals and more EM images.]

Response: We appreciate the suggestions of the reviewers. We have supplemented more HE images and TEM images of animals in PACE. For their locations, please refer to the newly submitted supplementary PDF file: "Supplement Raw Image - HE & TEM".

We believe the revised manuscript now addresses all concerns. We are grateful for the reviewers’ time and constructive feedback. Please do not hesitate to contact us if further revisions are required.

Sincerely,

Qingshan Ye

People's Hospital of Ningxia Hui Autonomous Region,Ningxia Medical University

No.301 Zhengyuan North Street, Yinchuan, Ningxia,China,750001

E-mail:yeqingshan@hotmail.com

22/02/2025
